# What is the “safe zone” for transition of coronal alignment from systematic to a more personalised one in total knee arthroplasty? A systematic review

**DOI:** 10.1007/s00167-021-06811-5

**Published:** 2022-01-01

**Authors:** Benjamin L. Schelker, Andrej M. Nowakowski, Michael T. Hirschmann

**Affiliations:** 1 Department of Orthopaedic Surgery and Traumatology, Kantonsspital Baselland (Bruderholz, Liestal, Laufen), CH-4101 Bruderholz, Switzerland; 2 Department of Clinical Research, Research Group Michael T. Hirschmann, Regenerative Medicine and Biomechanics, University of Basel, CH-4001 Basel, Switzerland

**Keywords:** Total knee arthroplasty, Coronal alignment, Kinematic alignment, Phenotype alignment, Personalised medicine, Safe zone

## Abstract

**Purpose:**

In total knee arthroplasty (TKA), implants are increasingly aligned based on emerging patient-specific alignment strategies, such as unrestricted kinematic alignment (KA), according to their constitutional limb alignment (phenotype alignment), which results in a large proportion of patients having a hip-knee angle (HKA) outside the safe range of ± 3° to 180° traditionally considered in the mechanical alignment strategy. The aim of this systematic review is to investigate whether alignment outside the safe zone of ± 3° is associated with a higher revision rate and worse clinical outcome than alignment within this range.

**Methods:**

A systematic literature search was conducted in PubMed, Embase, Cochrane and World of Science, with search terms including synonyms and plurals for “total knee arthroplasty”, “alignment”, “outlier”, “malalignment”, “implant survival” and “outcome”. Five studies were identified with a total number of 927 patients and 952 implants. The Oxford Knee Score (OKS) and the WOMAC were used to evaluate the clinical outcome. The follow-up period was between 6 months and 10 years.

**Results:**

According to HKA 533 knees were aligned within ± 3°, 47 (8.8%) were varus outliers and 121 (22.7%) were valgus outliers. No significant differences in clinical outcomes were found between implants positioned within ± 3° and varus and valgus outliers. Likewise, no significant differences were found regarding revision rates and implant survival.

**Conclusion:**

The universal use of the “safe zone” of ± 3° derived from the mechanical alignment strategy is hardly applicable to modern personalised alignment strategies in the light of current literature. However, given the conflicting evidence in the literature on the risks of higher revision rates and poorer clinical outcomes especially with greater tibial component deviation, the lack of data on the outcomes of more extreme alignments, and regarding the use of implants for KA TKA that are actually designed for mechanical alignment, there is an urgent need for research to define eventual evidence-based thresholds for new patient-specific alignment strategies, not only for HKA but also for FMA and TMA, also taking into account the preoperative phenotype and implant design. It is of utmost clinical relevance for the application of modern alignment strategies to know which native phenotypes may be reproduced with a TKA.

**Level of evidence:**

IV.

## Introduction

To achieve the longest possible implant survival, the aim of mechanical alignment in total knee arthroplasty (TKA) is to load the implant as evenly as possible and consequently to align the leg as neutrally as possible. Target values for mechanical alignment are a hip-knee-ankle angle (HKA) of 180°, a mechanical femur angle (FMA) of 90° and a mechanical tibia angle (TMA) of 90°. A deviation of ± 3° from neutral limb alignment, i.e., 180°, is considered acceptable and is currently regarded as a safe zone for successful implant alignment [10, 27]. Bellemans et al. [4] have triggered a major discussion in the community of knee surgeons about the optimal alignment target. The research group was able to show that within the general population 32% of men and 17% of women have a so-called constitutional varus alignment and only 66% of the male knees and 80% of the female knees have the target neutral limb alignment within ± 3°. The introduction of the concept of categorising limb alignment into phenotypes according to the FMA, the TMA and the HKA by Hirschmann et al. [16] underlined the variety of the individual alignments of the knee and highlighted the importance of the alignment of the tibial and femoral components and argued against the sole consideration of the HKA in the assessment of knee alignment in TKA. This is because although the majority of patients have a constitutionally neutral HKA, the knee phenotype, which represents the target of mechanical alignment not only in terms of HKA but also in terms of TMA and FMA, was found in only 5.6% of men and 3.6% of women [16]. Considering that forcing neutral alignment in patients with constitutional varus or valgus limb alignment requires significant soft tissue release, it is hypothesised that that forcing the knee into a position that is contrary to the constitutional alignment could be one of the major reasons for the persistently high level of dissatisfaction among one fifth of patients who have undergone TKA [7]. To restore an individual alignment that corresponds to the constitutional alignment of the knee before the onset of degenerative changes, unrestricted kinematic alignment was proposed by Howell et al. [19]. This alignment strategy involves tibial and femoral resections corresponding to the thickness and alignment of the components. As a result, soft tissue can be preserved and the need for ligament release can be minimised. Howell, unlike the supporters of restricted kinematic alignment, does not set restrictions regarding the preoperative anatomy of the patient and the postoperative alignment. Proponents of MA and restricted KA are therefore concerned that alignment outside a safe zone of ± 3° might lead to premature implant failure and argue that more severe limb alignment deviations from the neutral axis are biomechanically inferior and not compatible with current TKA implant designs [2].

The aim of this review is therefore to investigate (1) whether thresholds for postoperative alignment of HKA, TMA and FMA in modern TKA can be defined based on their impact on revision rates, implant survival and clinical outcome from existing literature and (2) whether the native knee phenotype of patients eventually has an impact on these thresholds.

## Materials and methods

A systematic literature search was conducted on PubMed, Embase, Cochrane and World of Science from their inception until May 18, 2021, to identify potentially relevant articles. Search terms including synonyms and plurals for TKA, alignment, implant positioning, kinematic, mechanical, anatomical, outlier, malalignment, varus, valgus, implant survival or outcome. The terms were linked with Boolean operators AND or OR and were searched for in both titles and abstracts. Inclusion criteria were English or German language publications in peer-reviewed journals comparing the outcome of different postoperative alignment phenotypes and outliers outside the traditional safe zone of hip-knee-ankle angle (HKA) ± 3° with the outcome of implants positioned with unrestricted kinematic alignment within the safe zone of ± 3°. Not original research or studies treating alignment strategies other than unrestricted kinematic alignment, revision TKA, osteotomies, unicompartmental knee arthroplasty, bicompartmental arthroplasty were excluded. Only full-text articles were included. After collecting all articles and removing duplicates, the studies were screened for inclusion criteria by title and abstract (BLS). In a second step, the selected articles were checked for their eligibility by full text analysis. In case of uncertainty regarding inclusion eligibility, a second author was consulted (MTH). Subsequently, the reference list of articles that met the above criteria were manually searched for further studies that were not covered by the original search terms. Endpoints included various patient-reported outcome measures (PROMs), implant survival and revision frequency in relation to different postoperative orientations (varus, neutral, valgus).

### Quality assessment

The methodological quality of the included studies was assessed using the Methodological Index for Non-Randomised Studies (MINORS) for non-randomised comparative and non-comparative clinical intervention studies [43]. The level of evidence of the included studies was reported.

### Data extraction

From the selected publications, the author (BS) extracted title, author, year of publication, study design, level of evidence, number of knees in each study group, implant type and surgery procedure, follow-up time, patient demographics, clinical outcome scores, radiological alignment into a Microsoft Excel spreadsheet.

### Statistical analysis

Continuous variables were described with means and standard deviations or medians and ranges. Categorical variables were reported with absolute and relative frequencies. For the interpretation of the data, a *p* < 0.05 was considered statistically significant.

## Results

### Search results and characteristics of included studies

**Fig. 1:**
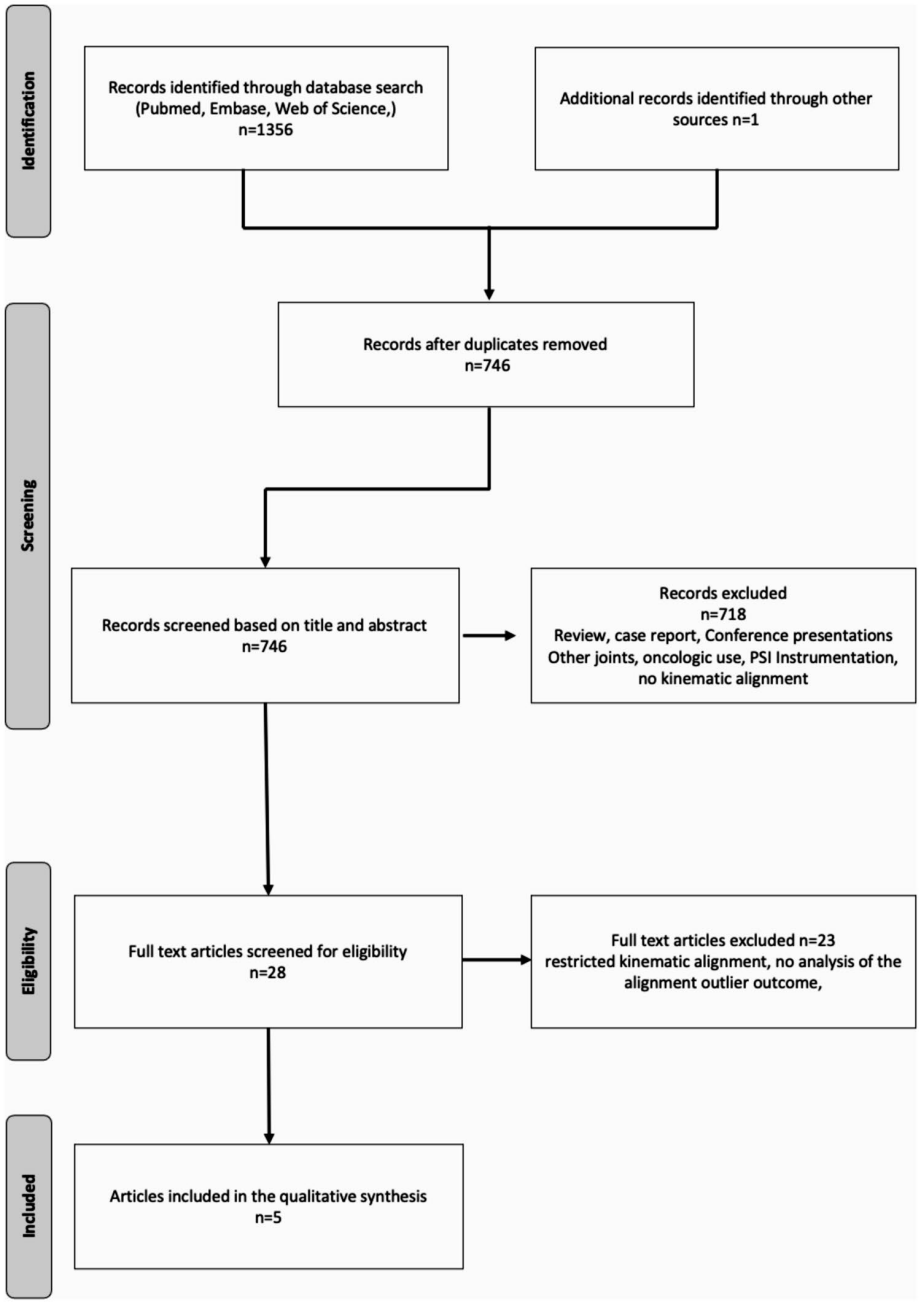
Flow-chart of the study selection process according to the PRISMA statement for the conduct of systematic reviews [33]

The literature search identified 1356 publications in the initial screening process. 5 of them met all the inclusion criteria following the process shown in Fig. 1. Detailed characteristics of the included studies are presented in Table 1. The mean MINORS score was 12 (SD ± 1.41).

**Table 1:** Overview selected studies

Author (year)	Number of patients (knees)	KA method	Age, years	Gender, male (%)	BMI, kg/m^2^,	Follow-up time, months	Level of evidence	MINORS score
Howell (2013) [19]	198 patients (214 knees)	PSI, CR	68 (36–95)	40%	30 (18–45)	38 (31–43)	IV	13
Howell (2013) [18]	101 patients (101 knees)	CI, CR	67 (8.9)	45%	30 ± 4.9	Minimum 6 (6–9)	IV	13
Howell (2015) [20]	214 patients (219 knees)	PSI CR	68 (10.1) 39–93	38%	31 ± 6.6 (14–49)	75.6 (69.6–86.4)	III	13
Howell (2018) [21]	216 patients (220 knees)	PSI CR	77 ± 10 (49–97)	37%	31 ± 6.1 (14–49)	120	III	11
Howell (2021) [17]	198 (198 knees)	Calipered, CR	67 ± 8	38%	29 ± 5 (18– 43)	47 ± 8 (33–66)	III	10

*PSI* patient-specific instruments, *CI* conventional instruments, *CR* cruciate retaining

### Revisions

Table 2 shows implant survival, revision rates overall and in relation to postoperative alignment, i.e., valgus, neutral or varus alignment.

**Table 2:** Revisions, reoperations and implant survivorship

Author (year)	Total (%)	In-range 0 ± 3°	Varus outliers > 3°		Valgus outliers < − 3°
Revisions during the study period (%)					
Howell (2013) [19]	0				
Howell (2013) [18]	0				
Howell (2015) [20]	5 (2.4%)				
Howell (2018) [21]	5 (2.5%)	3^a^ (2%)			
Howell (2021) [17]	1 (0.5%)			1	
Reoperations with retention of the components (%)					
Howell (2013) [19]	3 (1.4%)				
Howell (2013) [18]	0				
Howell (2015) [20]					
Howell (2018) [21]	2				
Howell (2021) [17]	2 (3 of 198)			2	
Implant survivorship in % (at years)					
Howell (2013) [19]	99.5% (2.6y)				
Howell (2013) [18]	100% (0.5y)				
Howell (2015) [20]	97.5% (6y)				
Howell (2018) [21]	97.4% (10y)	97.8% (10y)	100% (10y)	100% (10y)	
Howell (2021) [17]					

^a^TKAs with aseptic revisions

### Clinical outcomes regarding the alignment of the knee

The preoperative scores of the patients included in the studies are shown in Table 3. The Knee Society Score (KSS) and Oxford Knee Score (OKS) were recorded to assess the condition of the preoperative knee. The postoperative outcomes are shown in Table 4. According to the HKA, 533 knees were aligned within ± 3°, 47 (8.8%) varus outliers > 3° and 121 (22.7%) valgus outliers < − 3°. Looking at the tibia, 195 (21.7%) implants were aligned > 90° and 704 (78.3%) < 90° with respect to the mechanical axis. The patients in the 2021 study cannot be assigned to the traditional safe zone ± 3° because they were classified according to the phenotypes introduced by Hirschmann et al. All five included studies showed no statistically significant differences in postoperative clinical outcome scores measured by the Western Ontario and McMaster Universities Osteoarthritis Index (WOMAC) and OKS depending on alignment within ± 3°, varus or valgus alignment.

**Table 3:** Preoperative values

Author (year)	KSS pre^b^	KSS Function pre^b^	OKS pre^b^	Preoperative limb alignment^a, c^
Howell (2013) [19]	40 (14.5)	45 (19.4)	20 (7.9), 0–36	− 2° ± 7.9° (− 20° to 10°)
Howell (2013) [18]			22 (8.4), 0–36	− 2° ± 12.9° (− 30° to 20)°
Howell (2015) [20]			18 (7) 4–39	− 1.1° ± 6.3° (− 20° to 14°)
Howell (2018) [21]			18 (7) 4–39	− 1° ± 6.2° (− 20° to 14°)
Howell (2021) [17]	32 ± 12 (7–90)			− 1° ± 7° (− 17° to 14°)

^a^Varus ( +)/valgus ( −)

^b^Mean (SD)

^c^Mean SD (range)

**Table 4:** Outliers vs outcome

Author (year)	In range		Varus outliers		Valgus outliers	
	OKS	WOMAC	OKS	WOMAC	OKS	WOMAC
Tibial component alignment according to Ritter [32]	≥ 90°		< 90°			
Howell (2013) [19]	*N* = 49 (25%)		*N* = 143 (75%)			
	43 (41 to 44)	91 (88 to 95)	44 (42 to 45)	93 (90 to 95)		
Howell (2013) [18]	*N* = 4 (4%)		*N* = 96 (96%)			
	44 ± 3	95 ± 3	42 ± 5	89 ± 11		
Howell (2015) [20]	*N* = 43 (20%)		*N* = 168 (80%)			
	42 (40–45)	91 (86–95)	43 (41–44)	91 (89–93)		
Howell (2018) [21]	*N* = 42 (21%)		*N* = 156 (79%)			
	42 (40–45)	93 (86–95)	43 (41–44)	91 (91–95)		
Howell (2021) [17]	*N* = 57 (29%)^a^		*N* = 141 (71%)^b^			
	OKS was not significantly different between the TMA phenotypes					
Limb alignment (HKA) according to Paratte [38]	0° ± 3°		> 3°		< 3°	
Howell (2013) [19]	*N* = 141 (73%)		*N* = 11 (6%)		*N* = 40 (21%)	
	43 (42 to 45)	92 (90 to 94)	47 (43 to 50)	99 (91 to 107)	43 (41 to 45)	92 (88 to 95)
Howell (2013) [18]	*N* = 93 (93%)		*N* = 6 (6%)		*N* = 1 (1%)	
	42 ± 5	89 ± 11	44 ± 3	95 ± 5	38	
Howell (2015) [20]	*N* = 154 (73%)		*N* = 15 (7%)		*N* = 42 (20 = %)	
	43 (41–44)	91 (89–94)	42 (38–46)	92 (85–99)	42 (40–45)	89 (85–94)
Howell (2018) [21]	*N* = 145 (73%)		*N* = 15 (8%)		*N* = 38 (19%)	
	44 (43–45)	93 (91–95)	45 (41–48)	97 (94–99)	41 (39–44)	88 (83–94)
Howell (2021) [17]						
	OKS was not significantly different between the HKA phenotypes					

^a^Valgus TMA phenotypes according to [15]: VAL_TMA_6°: *N* = 4 (2%) VAL_TMA_3°: *N* = 53 (26.8%)

^b^Neutral and varus TMA phenotypes according to [15]: NEU_TMA_0° *N* = 111 (56.1%) VAR_TMA_3° *N* = 29 (14.6%) VAR_TMA_6° *N* = 1 (0.5%)

## Discussion

The main findings of this study are that both the clinical outcome and revision rates of patients with unrestricted KA-TKA appear to be independent of a postoperative alignment deviation of more than 3° from both the tibial component 90° and the whole limb alignment 180°. However, it is not possible to determine from the available data whether there is a safe zone for TKA alignment and where this safe zone might be located. Although 31.5% of patients have an alignment outside the traditional safe zone of ± 3°, these were not further analysed for their specific phenotypes and clinical outcomes.

The finding that alignment within the traditional safe zone does not provide clinical benefit contradicts the results of older studies from 1977–2019 [5, 8, 10, 24, 28, 37, 39, 45], which demonstrated a correlation between deviation of more than ± 3° and worse clinical outcome, but supports the results of more recent long-term studies in mechanically aligned implants [1, 6, 23, 29, 31, 34, 38]], which also demonstrated no correlation between alignment and outcome. The first study to report a correlation between a good clinical outcome and optimal neutral alignment was the study by Lotke et al. in 1977 [28]. Larger and more recent studies by Fang et al. [10] in 2009, Ritter et al. [39] in 2011, Kim et al. [24] in 2014 and Park et al. [37] in 2018 showed higher revision rates in the postoperative outlier groups of ± 3°. In particular in the case of a varus alignment of the tibial component, higher revision rates were found than with neutrally aligned implants (3.8% vs 0.2%, Ritter [39]) and (3.4% vs 0%, Kim [24]). According to the results of the radiostereometric analysis (RSA) study by Teeter et al. [44], one reason for the increased failure of varus-aligned implants could be increased component migration in varus-aligned tibial implant. However, the study failed to demonstrate a correlation between increased migration and overall limb alignment. The results of van Hamersveld et al. [46] also indicate a significantly increased migration of the tibial component in mechanically varus aligned TKAs. However, in another RSA study by Laende et al. [26], no difference was found between component migration in KA-TKA and MA-TKA, and no correlation was shown between migration and alignment. This is consistent with the findings of studies showing no difference in revision rates between in-range and outliers MA-TKAs. Several authors [1, 6, 22, 23, 29, 30, 34, 38] with follow-up periods of up to 20 years reported no association between revision rates for implants within the ± 3° safe zone and outliers. Abdel et al. [1], Kathib et al. [22] and Salzmann et al. [40] compared clinical outcome using KSS and WOMAC between patients with implants aligned within ± 3° and outliers and found no significant difference. Another study whose results contradict the assumption that correcting the mechanical alignment to ± 3° from 180° leads to the best results after TKA is the study by Vanlommel [48], which showed that patients with preoperative varus alignment after KSS show a better outcome when left in mild varus. However, the extent of remaining varus alignment seems crucial, as the study [48] was able to show that a varus alignment of > 6° leads to a worse clinical outcome.

When looking at the existing literature on alignment in TKA, two issues stand out. Firstly, different radiological methods were used to measure alignment, and secondly, the studies aimed at mechanically aligned TKAs compared unintentional outliers with well-aligned TKAs. Hence, in most studies where an adverse correlation between outlier group and revision rates has been found so far, short leg radiographs were used to determine limb alignment. In contrast, in the studies where no difference was found, only full-leg radiographs were used. Indeed, several studies have shown that short radiographs of the knee, in contrast to full-leg radiographs, are of limited value in determining HKA [11, 13, 41, 47]. An indication that it makes a difference whether an implant is intentionally implanted outside the ± 3° safety zone or whether it is an unintentional malposition is found in the already cited study by van Hamersveld [46]. Although there was increased component migration in the varus group, the difference between varus, in-range and valgus disappeared when the orientation of the component was matched to the constitutional orientation. It has also been shown that with kinematically aligned TKA, the intraoperative forces in the medial and lateral compartments in patients with outlier alignment were comparable to those with in-range alignment, with no evidence of overloading of the medial or lateral compartment of the knee [42].

The fact that the risk of failure of the tibial component is not increased after 10 years is explained by Howell, the pioneer of KA-TKA, with KA-TKA restoring the native joint line, providing a more physiological strain on the collateral ligaments. [9], with the balancing of the medial and lateral ligament structures without overloading the lateral or medial compartment [42] and the reduced adduction moment during gait [36].

The answer to the question regarding the influence of the preoperative alignment on the clinical outcome and on a possible safe zone cannot be answered on the basis of the available data, since the influence of the preoperative alignment on the revision rate was only investigated in one of the included studies [17]. In this study, it was found that patients who suffered from patellofemoral pain after KA-TKA had a more valgus limb axis preoperatively. However, the authors of the study mainly blame the design of the femoral component for the patellofemoral problems, as it was not designed for valgus alignment and the trochlea would be too narrow for a more valgus alignment [17]. Yet, the question to what extent the implant design has an influence on the definition of a safe zone for patient-specific alignment strategies cannot be answered with the present Systematic Review.

There are several limitations to this study.

First of all, all papers included in this review were written by the same author. This author is also the inventor of the KA-TKA technique and is therefore prone to bias, as it is known that the developer of a technique often achieves significantly better results than independent registry studies using the same technique [3, 25]. It is also not entirely clear to what extent the patients described in the different studies are identical. Other limitations of the included studies are that four out of five studies only compared the clinical outcome with the HKA and the TMA without considering the FMA. Another important limitation of this study is the restriction of the examination to the coronal plane, as component malalignments in the sagittal plane can have an impact on the flexion pattern and axial malalignments can lead to patellar malalignments and anterior knee pain [12, 14, 35]. In addition, it should be noted that none of the included studies provided information on a sample size analysis. It must also be taken into account that the follow-up period of the studies was very heterogeneous.

For day-to-day clinical practice, the results of this study show that as long as no generally accepted guidelines for phenotype-specific alignment of implants have been established by the orthopaedic community, coronal implant alignment has to be determined on a case-by-case basis, not so much on the basis of a rigid number of degrees, but rather on the basis of several factors such as the implant model, the surgical technique, the patient's phenotype, the soft tissue situation and, last but not least, the surgeon's skills and experience.

## Conclusions

The universal use of the “safe zone” of ± 3° derived from the mechanical alignment strategy hardly applicable to more modern alignment strategies in the light of current literature. However, given the conflicting evidence on the risks of higher revision rates and poorer clinical outcomes with greater tibial component deviation, the lack of data on the clinical outcomes of more extreme alignments and the use of implants designed for mechanical alignment, there is an urgent need for research to define eventual evidence-based thresholds for new patient-specific alignment strategies.
